# Hypoxia promotes the migration and invasion of human hepatocarcinoma cells through the HIF-1α–IL-8–Akt axis

**DOI:** 10.1186/s11658-018-0100-6

**Published:** 2018-09-20

**Authors:** Maoyun Fei, Jianming Guan, Tao Xue, Lianjin Qin, Chengwu Tang, Ge Cui, Yao Wang, Hui Gong, Wenming Feng

**Affiliations:** 1 Department of General Surgery, The First People’s Hospital of Huzhou, No.158 Guangchanghou Road, Zhejiang Province 313000 Huzhou, People’s Republic of China; 2 Department of Ultrasound, The First People’s Hospital of Huzhou, No.158 Guangchanghou Road, Zhejiang Province 313000 Huzhou, People’s Republic of China; 3 Central Laboratory, The First People’s Hospital of Huzhou, No.158 Guangchanghou Road, Zhejiang Province 313000 Huzhou, People’s Republic of China; 4 Department of Hepatobiliary Pancreatic Surgery, The First People’s Hospital of Huzhou, No.158 Guangchanghou Road, Zhejiang Province 313000 Huzhou, People’s Republic of China

**Keywords:** Hepatocarcinoma, Hypoxia, HIF-1α, IL-8, Akt pathway

## Abstract

**Background:**

Hepatocellular carcinoma (HCC) is the fifth most common cancer and the third most common cause of cancer-related death worldwide. The 5-year survival rate remains low despite considerable research into treatments of HCC, including surgery, radiotherapy and chemotherapy. Many mechanisms within HCC still require investigation, including the influence of hypoxia, which has a crucial role in many cancers and is associated with metastasis. Hypoxia inducible factor-1α (HIF-1α) is known to regulate the expression of many chemokines, including interleukin-8 (IL-8), which is associated with tumor metastasis. Although many studies have reported that HIF-1α is associated with HCC migration and invasion, the underlying mechanisms remain unknown.

**Methods:**

The expression level of HIF-1α was determined in HCC cells. The correlation of IL-8 and HIF-1α expressions was assessed via knockdown of HIF-1α. HCC cells were also used to assess the influence of HIF-1α on HCC cell migration and invasion. LY294002, an inhibitor of the Akt pathway, was used to confirm the associated signaling pathways.

**Results:**

We observed a significant attenuation of cell migration and invasion after silencing of HIF-1α. Exogenously expressing IL-8 restored migration and invasion. Akt was found to be involved in this process.

**Conclusion:**

Hypoxia promotes HCC cell migration and invasion through the HIF-1α–IL-8–Akt axis.

## Background

Hepatocellular carcinoma (HCC) is the fifth most common cancer and the third most common cause of cancer-related death worldwide [1]. Although advances have been made in diagnostic and treatment strategies, the 5-year survival rate remains low because of the high rates of metastasis [2, 3]. Several pathogenic mechanisms and factors associated with HCC have been documented, but the molecular mechanisms of HCC migration and invasion still need investigation [4].

Previous studies showed that hypoxia promotes metastasis by inducing hypoxia inducible factor-1 (HIF-1) [5–7]. HIF-1 consists of two subunits: HIF-1β: a constitutively expressed subunit; and HIF-1α, an activity-determining unit that regulates tumor metabolism, proliferation and metastasis [8–10]. Recent studies showed that HIF-1α has a role in HCC cell migration and invasion [11–13]. This concurs with our finding that HCC cell migration and invasion are sharply attenuated by knockdown of HIF-1α under conditions of hypoxia. However, the underlying mechanisms remain largely unknown.

It is known that HIF-1α can stimulate the expression of various cytokines and chemokines [14–17]. Interleukin-8 (IL-8) is a chemokine with tumorigenic properties. It is associated with tumor metastasis in several cancer types [18–20]. A previous study illustrated that cells can produce IL-8 in response to hypoxia [21]. IL-8 was also recently reportedto be co-expressed with HIF-1α in HCC with this co-expression is associated with metastasis and poor prognosis in HCC [11].

In this study, we found that IL-8 is regulated by hypoxia induced-HIF-1α and that it can restore HCC cell migration and invasion attenuated by knockdown of HIF-1α. This suggests a correlation between HIF-1α and IL-8 expression and a significant role for this correlation on HCC cell migration and invasion.

The Akt signaling pathway is one of the key mechanisms of tumor survival. It has the ability to promote metastasis [22]. A recent study demonstrated that IL-8 promotes the invasion of human osteosarcoma cells through the Akt signaling pathway [23]. Here, we observed that the addition of an Akt pathway inhibitor decreased HCC cell migration and invasion, while exogenous expression of HIF-1α prevented this decrease. Our conclusion is that HIF-1α promotes HCC cell migration and invasion through the IL-8–Akt axis.

## Materials and methods

### Cell cultures

The human HCC cell lines Hep3B andHuh7 and the normal liver cell line WRL68 were obtained from the Shanghai Institute of Biological Sciences of the Chinese Academy of Sciences. The cells were cultured in Dulbecco’s modified Eagle’s medium (DMEM) supplemented with 10% fetal bovine serum (FBS;GIBCO-BRL) in a humidified atmosphere of 95% normal air and 5% CO_2_ at 37 °C. For the hypoxia experiments, the cells were incubated in a humidified HetoMulti-gas incubator with an atmosphere of1% O_2_, 5% CO_2_ and 94% N_2_.

### RNA isolation and quantitative RT-PCR

Total RNA was extracted from the cells using the Trizol reagent (Invitrogen) according to the manufacturer’s protocol. Reverse transcription was performed using a PrimeScript RT Reagent Kit (TaKaRa). For quantitative RT-PCR, cDNA was amplified using SYBR Premix Ex Taq (TaKaRa). Glyceraldehydes-3-phosphate dehydrogenase (GAPDH) was used as a control and the experiments were performed in triplicate. The primer sequences were:

- HIF-1α sense, 5’-GAACGTCGAAAAGAAAAGTCTC-3’
- HIF-1α antisense, 5’-CCTTATCAAGATGCGAACTCACA-3’
- IL-8 sense, 5’-CAGCCTTCCTGATTTCTGC-3’
- IL-8 antisense, 5′-GGGTGGAAAGGTTTGGAGTA-3’
- GAPDH sense, 5’-TGACTTCAACAGCGACACCCA-3’
- GAPDH antisense, 5’-CACCCTGTTGCTGTAGCCAAA-3’

### Western blot analysis

Total cell lysates were subjected to 10% SDS-PAGE and the proteins were transferred to nitrocellulose filter membranes, followed by blocking for 1 h in 5% non-fat dry milk. The membranes were incubated with primary antibodies at 4 °C overnight, and then with secondary antibodies at room temperature for 2 h. GAPDH was used as a gel loading control.

### Cell migration and invasion assays

For the migration assay, transwell chambers (Corning) with 8-μm pore size polycarbonate filter inserts for 24-well plates were used according to the manufacturer’s instructions. 1 × 10^5^ ells were seeded onto the upper compartment in 200 μl DMEM with 0.1% FBS followed by placement into wells containing 500 μl complete medium in the lower chamber for 24 h at 37 °C. Then, the cells on the upper surface of membrane were removed and the cells attached to the lower surface of membrane were fixed and stained with Giemsa stain. The number of cells was counted under an inverted microscope.

Invasion was assayed using the same procedure as the migration assay, except that 70 μl of 1 mg/ml Matrigel (BD Biosciences) was added to the upper face of the membrane. Assays were repeated three times.

### Small interfering RNA (siRNA) and transient transfection

siRHIF-1α was generated with the sequence 5’-GUGAUGAAAGAAUUACGAAUTT-3′ (sense) and 5’-AUUCGGUAAUUCUUUCAUCACTT-3′ (antisense), used to generate pSilencer3.1–HIF-1α as previously described [11]. The scrambled sequences 5’-UUCUCCGAACGUGUCACGUdTdT-3′ and 5’-ACGUGACACGUUCGGAGAAdTdT-3′ were produced. siRNA transfection was performed using Lipofectamine 3000 (Invitrogen).

### Chromatin immunoprecipitation (ChIP) assay

Anti-HIF-1α antibody (R&D Systems) was used to immunoprecipitate sonicated chromatins prepared from HCC cell lines. IgG was used as a control. Immunoprecipitated DNA was quantified for the IL-8 promoter segments using real-time PCR. The primer sequences were:

- Forward primer, 5’-CCCTCGAGCATACTCCGTATTTGATAAGGAAC-3’
- Reverse primer, 5’-GGCTCTTGTCCTAGAAGCTT-3’

### Statistical analysis

All data are presented as means ± SD (standard deviation). Differences between groups were tested for statistical significance using Student’s *t-*test or χ^2^-test. *p* < 0.05 was considered statistically significant.

## Results

### The high expression level of HIF-1α in HCC under conditions of hypoxia

**Fig. 1:**
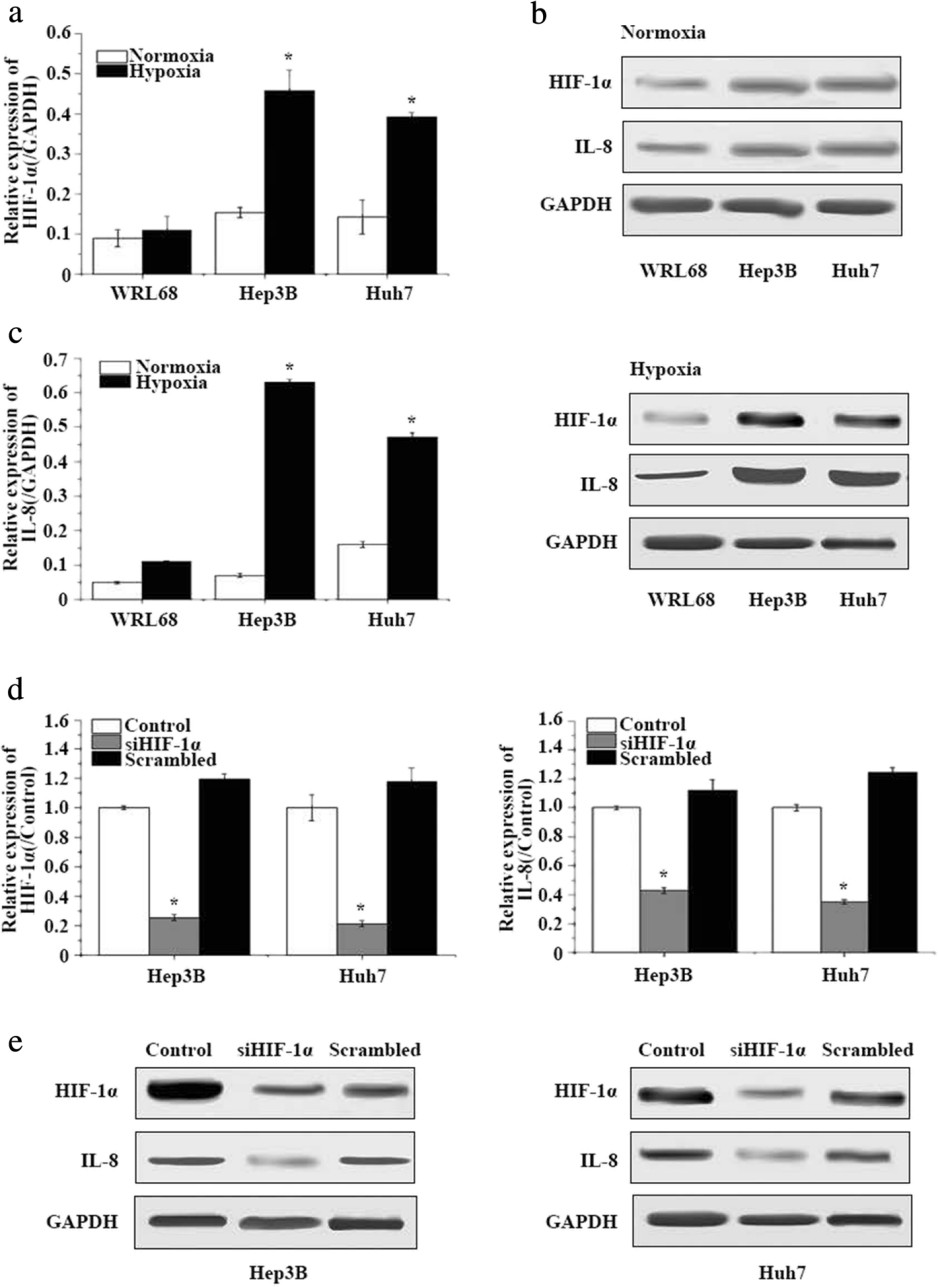
HIF-1α is associated with the migration and invasion of human hepatocarcinoma (HCC)cells by regulating IL-8 expression under conditions of hypoxia*.* **a** Relative HIF-1α mRNA levels in human HCC cell lines and the normal liver cell line WRL68. *n* = 3; *p < 0.05. **b** HIF-1α and IL-8 protein levelsin HCC cell lines and the normal liver cell line WRL68. GAPDH was used as the loading control. **c** Relative IL-8 mRNA levels in human HCC cell lines and the normal liver cell line WRL68. *n* = 3; *p < 0.05. **d** The efficiency of HIF-1α knockdown in HCC cell lines (left panel) and the effect on IL-8 expression (right panel) were evaluated using quantitative RT-PCR. **e** Western blot analysis of HIF-1α and IL-8 protein expression in HCC cell lines treated with siHIF-1α. GAPDH was used as a control. *n* = 3; *p < 0.05

Quantitative RT-PCR was performed to assess the expression level of HIF-1α in the human HCC cell lines Hep3B and Huh7 and the control liver cell line WRL68 under conditions of hypoxia and normoxia. The mRNA level of HIF-1α was significantly higher in both HCC cell lines than in WRL68 under hypoxia, while there were no statistical differences between the HIF-1α expression levels for the three cell lines under normoxia (Fig. 1a). The results of the western blot assay concurred with this result (Fig. 1b). This suggests that hypoxia may promote HIF-1α expression in HCC cell lines. The expression profiles of IL-8 in the HCC cell lines and normal liver cells also correlate with those of HIF-1α (Fig. 1 b and c).

### HIF-1α is associated with migration and invasion of HCC through the regulation of IL-8 expression under conditions of hypoxia

**Fig. 2:**
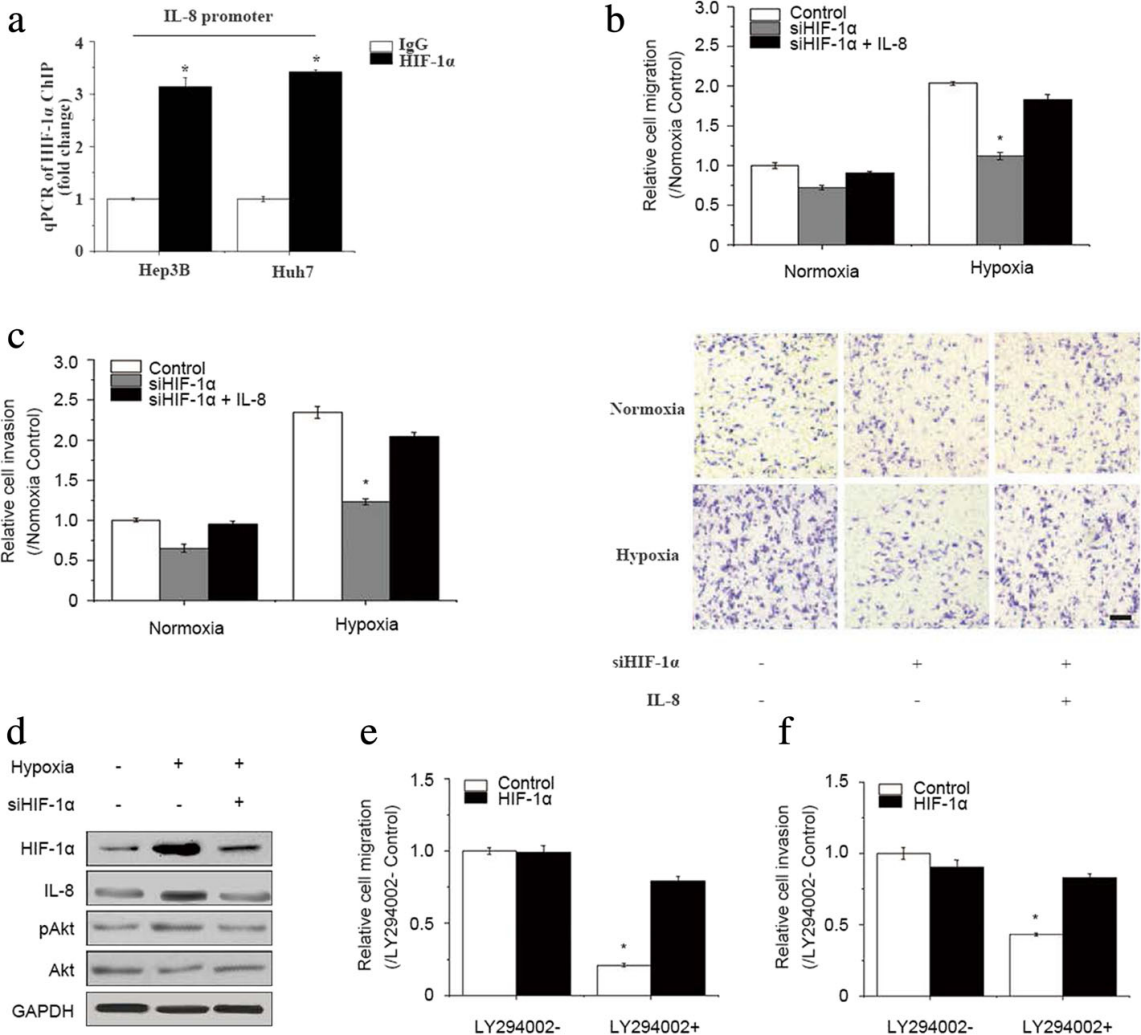
Exogenous IL-8 restored the migration and invasion that had been attenuated by Akt pathway inhibition*.* **a** HIF-1α antibody was used for ChIP, followed by quantitative PCR with specific primer pairs for the IL-8 promoter (*n* = 3; *p < 0.05) in two HCC cell lines under hypoxia. **b** Relative Hep3B cell migration aftersiHIF-1α treatment and IL-8 addback treatment under normoxia and hypoxia. The numbers of migrated cells from 5 independent experiments were analyzed. Scale bar: 200 μm (bottom panel) **c** Relative Hep3B cell invasion aftersiHIF-1α treatment and IL-8 addback treatment under normoxia and hypoxia. **d** Western blot assay of HIF-1α, IL-8, pAkt and the Akt response to siHIF-1α treatment. GAPDH was used as the loading control. **e** Relative Hep3B cell migration showed a decrease when cells overexpressing HIF-1αwere treated with LY294002. **f** – Relative Hep3B cell invasion showed a decrease when cells overexpressing HIF-1α were treated with LY294002. All results represent three independent experiments. LY294002-: treated without LY294002; LY294002+: treated with LY294002

siHIF-1α was introduced to silence HIF-1α, and was confirmed effective at the mRNA level (Fig. 1d, left panel) and protein level (Fig. 1e) in both HCC cell lines under hypoxia. In addition, compared with the control and scrambled groups, at both the mRNA level (Fig. 1d, right panel) and protein level (Fig. 1e), IL-8 was significantly downregulated by the silencing of HIF-1α in both HCC cell lines, suggesting that IL-8 expression may be regulated by HIF-1α. ChIP-qRT-PCR was performed using an anti-HIF-1α antibody or an IgG control in both HCC cell lines under conditions of hypoxia. The results showed that HIF-1α directly associated with the IL-8 promoter region, confirming that IL-8 was activated by HIF-1α in HCC cells under hypoxia (Fig. 2a).

The roles of HIF-1αin HCC cell migration and invasion were assessed using the transwell assay. siHIF-1α was transfected into Hep3B cells under hypoxia or normoxia. With conditions of hypoxia, the number of migrated and invaded cells sharply decreased when HIF-1α was silenced (420 vs. 232 migrated cells per field and 524 vs. 276 invaded cells per field). This was not observed under normoxia (Fig. 2b and c, 207 vs. 149 migrated cells per field and 224 vs. 146 invaded cells per field). This implies the elimination of HIF-1α-attenuated HCC cell migration and invasion under hypoxia. Overexpression of IL-8 restored the migratory and invasive capacities of HCC cells transfected with siHIF-1α (Fig. 2b and c, from 149 to 188 migrated cells and from 276 to 457 invaded cells per field). Therefore, IL-8 expression may be regulated by HIF-1α and HIF-1α may promote HCC cell migration and invasion by regulating IL-8 expression under conditions of hypoxia.

### Exogenous HIF-1α restored the migration and invasion that was attenuated by Akt pathway inhibition

A recent study demonstrated that IL-8 promotes the invasion of human osteosarcoma cells through the Akt signaling pathway. This pathway is associated with metastasis in many tumor types. We hypothesized that the effect of HIF-1α on HCC cell migration and invasion could be associated with IL-8-induced Akt pathway activation. To confirm this hypothesis, we transfected siHIF-1α into Hep3B cells under conditions of hypoxia or normoxia, and then determined the pAkt expression level. The results show that the expression level of IL-8 decreased after HIF-1α silencing under hypoxia. In addition, the protein level of pAkt was simultaneously decreased (Fig. 2d), indicating that HIF-1α may regulate IL-8 and thereby promote Akt pathway activation.

To further investigate the role of the HIF-1α–IL-8–Akt axis on HCC cell migration and invasion, the Akt inhibitor LY294002 was introduced. Hep3B cells were treated with 10 μM LY294002 followed by a transwell assay to assess the number of migrated and invaded HCC cells under hypoxia. The results showed an obvious decrease in the relative migration and invasion of HCC cells after Akt pathway inhibition (Fig. 2e and f). However, exogenous expression of HIF-1αsignificantly increased the number of migrating and invading HCC cells compared to the group without LY294002 treatment. Therefore, hypoxia may promote HCC cell migration and invasion through the HIF-1α–IL-8–Akt axis.

## Discussion

Hypoxia plays an important role in many cancers and hypoxia-inducible factors are crucial mediators of the hypoxia response [14]. In cancer, intratumoral hypoxia promotes tumor aggressiveness, increasing metastasis and patient mortality. The activation of HIF-1α is known to play a role in this [24]. Previous studies have reported that hypoxia promotes HCC invasion and progression and that overexpression or stabilization of HIF-1α is involved [25, 26]. This is consistent with our results showing that HIF-1α was highly expressed in HCC cells under conditions of hypoxia and that hypoxia-induced HIF-1α promoted HCC cell migration and invasion.

HIF-1α regulates the expression levels of various chemokines and their receptors to promote the proliferation and metastasis of many cancers [27–29]. IL-8, also known as CXCL8, is a chemokine with a role in metastatic and advanced cancers. It promotes tumor growth, metastasis and angiogenesis [21, 30]. A recent study proved that co-expression of IL-8and HIF-1α is associated with metastasis and poor prognosis in hepatocellular carcinoma [11]. In this study, we found that the expression of IL-8 was regulated by HIF-1α in HCC cells under hypoxia, and that overexpression of IL-8 increased the number of migrated and invaded cells, even reversing the attenuation caused by HIF-1α silencing. This suggests that IL-8 is essential for HIF-1α-promoted HCC cell migration and invasion.

The Akt pathway is known to be involved in tumor metastasis [22], and a target of AKT, mTOR, is associated with promoting proliferation and differentiation, and inhibiting apoptosis [31]. A recent study showed that the Akt pathway is activated in HCC cells under hypoxia [11]. In this study, we observed that an Akt inhibitor, LY294002, attenuated HCC cell migration and invasion, but exogenous HIF-1αreversed these effects. Additionally, silencing of HIF-1α simultaneously downregulates the expression levels of IL-8 and Akt, suggesting a correlation betweenIL-8, HIF-1α and Akt. However, how the Akt pathway further promotes HCC cell migration and invasion and the mechanism of how HIF-1α regulates IL-8 were not covered in this study. Further research can focus on these questions.

## Conclusion

Hypoxia activates HIF-1α in HCC cells to regulate IL-8 expression, and thereby promotes HCC cell migration and invasion through the Akt pathway.

## Abbreviations

CXCL8: C-X-C motif ligand 8
HCC: Hepatocellular carcinoma
HIF-1α: Hypoxia inducible factor-1α
IL-8: Interleukin-8
